# Cremation

**Published:** 1875-03

**Authors:** 


Cremation.—The New York *Evening Post* published the following editorial, October 24, 1855. During the twenty years that have elapsed, the idea—which, on sanitary grounds, certainly is commendable—has gained adherents. As the argument is as sound now as then, and as the style is charming, we reproduce it:

"The burning of a dead body at Milwaukee, in Wisconsin, as a preliminary to sepulture, seems to have occasioned a good deal of excitement at that place, and one of the Milwaukee daily prints inveighs against the act as if it were a crime. We have never heard of any other example of such a mode of disposing of the dead in this country, by the act of man; although Providence sometimes sees fit, in the conflagrations which occur in cities and elsewhere, to permit human bodies to be reduced to ashes by fire.

"For our part, we are very far from viewing the subject in the light in which the Milwaukee print regards it. It seems to us that any mode of disposing of the remains of the dead, which is convenient, which is not prejudicial to the public health, nor inconsistent to the solemnity of death, is perfectly proper, and cannot be rightfully interfered with. At sea the practice is to attach weights to the dead, and sink them to the bottom, there to become the prey of the creatures of the deep. On the land they are placed in vaults, to be devoured by worms, or laid deep in the earth, to be resolved into the elements by a slower decomposition. Either of these methods has something in it at which the imagination revolts, and which invests death with certain physical horrors, diverting the mind from the more important aspect in which it should be regarded—the great spiritual change it brings with it. The process of cremation comes in, immediately after life has departed, to intercept chemical decomposition and the ravages of inferior living creatures. It resolves the body into the elements to which it must finally return, by a more rapid change, which is neither offensive to the senses nor shocking to the imagination. There is nothing in it incompatible with the reverence which the heart naturally yields to the spectacle of death, nor with the affection with which we regard the remains of our friends. The ancients gathered the ashes of the funeral pyre into an urn, which was as dear and as sacred to the surviving friends of the dead as a grave is now.

"The most civilized and polished nations of antiquity practiced the mode of cremation, and there are still many who hold that they were guided to it by the same good sense and fortunate combination of the mental faculties which achieved their civilization. How it happened that in embracing Christianity they laid this custom aside and adopted that of a ruder nation is easily explained. Their first Christian teachers, who conducted their funerals, were of the Jewish race, and would naturally prefer the mode of sepulture common in their native country.

"Another reason is sometimes given, namely, that the early Christian converts were in daily expectation of the end of the world, and of the resurrection, and could not think of consuming by fire a frame into which the breath of life would again be so soon breathed. They laid away in chambers of the rock those who had fallen asleep, in the hope of a speedy re-awakening. There is nothing, however, in the process of cremation inconsistent with any of the doctrines of Christianity, as the western journalist whose invectives we have quoted in this sheet strangely seems to suppose. If there be, what shall we do with the ashes of the martyrs and confessors of the Christian faith who have perished at the stake, and whose mode of death the church counts among her glories? Their ashes, and the ashes of those who from time to time are destroyed by the conflagration of dwellings, are as much in God's hand as the dust of those who decay in their coffins.

"If the practice of cremation had been general we suppose there is no doubt that the health of the large towns of the Old World would have been more secure than it has been for centuries past. Crowds of the dead, festering in church-yards and cemeteries, amidst inhabited dwellings and beside thronged streets, have been a potent cause of distempers, which is now generally recognized. The law interferes and bids the citizen bury his dead at a distance from the haunts of men, except, perhaps, at certain seasons of the year, when there is supposed to be less danger. Here there is a clear recognition of the common mode of burial as dangerous to public health. The ordinary process of animal decomposition is nauseous, unwholesome, infectious. Graves casually or purposely opened have smitten down with their exhalations those who opened them, and diffused pestilence. The process of decomposition by fire, on the other hand, is a cleansing and purifying process.

"We have put these considerations together as some antidote to the unreflecting intolerance which so violently condemns the example set the other day in Milwaukee. It is not an example which is likely to be followed, and the aid of legislation, which is invoked to prevent it from being copied, is quite unnecessary. Mr. Pfeil, when about to place the body of his wife, in compliance with her dying request, on the funeral pyre, remarked that there was no law in Wisconsin forbidding the act. We are not aware that the practice of cremation is against the law anywhere. The body of Shelley was burned on a funeral pyre in despotic Tuscany, without any interference from the authorities. If it be intended to attract attention to the practice and awaken inquiry and open the way for making it eventually a frequent one, the most effectual means will be to pass laws prohibiting it, for the question could hardly be investigated without some persons becoming persuaded that the ancient method of the Romans is most conformable to civilized ideas."

